# Insights into Dyslexia Genetics Research from the Last Two Decades

**DOI:** 10.3390/brainsci12010027

**Published:** 2021-12-26

**Authors:** Florina Erbeli, Marianne Rice, Silvia Paracchini

**Affiliations:** 1 Department of Educational Psychology, Texas A&M University, College Station, TX 77843, USA; marianne.rice@tamu.edu; 2 School of Medicine, University of St Andrews, St Andrews KY16 9AJ, UK; sp58@st-andrews.ac.uk

**Keywords:** dyslexia, genetics, twin studies, molecular genetic studies

## Abstract

Dyslexia, a specific reading disability, is a common (up to 10% of children) and highly heritable (~70%) neurodevelopmental disorder. Behavioral and molecular genetic approaches are aimed towards dissecting its significant genetic component. In the proposed review, we will summarize advances in twin and molecular genetic research from the past 20 years. First, we will briefly outline the clinical and educational presentation and epidemiology of dyslexia. Next, we will summarize results from twin studies, followed by molecular genetic research (e.g., genome-wide association studies (GWASs)). In particular, we will highlight converging key insights from genetic research. (1) Dyslexia is a highly polygenic neurodevelopmental disorder with a complex genetic architecture. (2) Dyslexia categories share a large proportion of genetics with continuously distributed measures of reading skills, with shared genetic risks also seen across development. (3) Dyslexia genetic risks are shared with those implicated in many other neurodevelopmental disorders (e.g., developmental language disorder and dyscalculia). Finally, we will discuss the implications and future directions. As the diversity of genetic studies continues to increase through international collaborate efforts, we will highlight the challenges in advances of genetics discoveries in this field.

## 1. Clinical Diagnosis and Educational Identification and Manifestation of Dyslexia

The term dyslexia, defined as a specific learning disability, was thought to be first used by Dr. Pringle Morgan in 1896. It is one of the oldest and most well-known terms associated with reading disabilities [1]. Dyslexia is characterized by difficulties with accurate and/or fluent word reading and poor spelling abilities. These difficulties are neurobiological in origin and result from a phonological processing deficit (i.e., the ability to hear, remember, and recall different sounds in speech) [2], which is unexpected based on other cognitive abilities and access to effective classroom instruction [3]. Dyslexia is typically identified and manifested in educational settings. Universally accepted operational criteria for identifying and assessing dyslexia do not exist, though, and differences among clinical and educational assessment procedures can mean inconsistencies in identification.

From a clinical perspective, the Diagnostic and Statistical Manual of Mental Disorders (DSM-5) lists four criteria that must be met to diagnose a specific learning disability, which includes dyslexia. They are as follows: (a) persistent difficulties (i.e., for 6 months or more) in reading, such as inaccurate or slow and effortful reading; (b) skills must be well below average for the person’s age and interfere with academic achievement or daily life; (c) difficulties that begin during the school-age years even though some people may not have significant problems until adulthood; and (d) difficulties are not better explained by another disorder [4].

From an educational perspective, criteria for dyslexia identification can vary significantly across schools and countries. Education researchers have generally shown support for a model of identification that considers dyslexia as a construct that is not associated with any specific, single criterion but a combination of criteria for identification. One such proposed model includes three criteria. It does not, however, fundamentally differentiate dyslexia from other specific learning disabilities in reading. This three-pronged approach is operationalized as the following: (a) low achievement in reading (specifically inaccurate or not fluent word reading or spelling); (b) poor response to effective instructional practices, including multi-tiered systems of support; and (c) exclusion of other factors (e.g., intellectual disability or attention-deficit/hyperactivity disorder) requiring additional evaluation [5]. Similarly, Wagner and colleagues [6] propose a hybrid model with a constellation of criteria to identify dyslexia, with an increased focus on the unexpectedness of the reading difficulties. Four criteria of dyslexia are considered: (a) unexpected poor phonological decoding of nonsense words (i.e., poorly applying knowledge about sound–letter correspondences when reading nonsense words); (b) unexpected poor sight word reading (e.g., poorly reading lists of common words for a student’s age, which should be recognized without sounding out the letters); (c) poor response to effective instruction, including multi-tiered systems of support; and (d) higher listening comprehension compared to reading comprehension. The similarities between the abovementioned models of identification include low achievement in reading (e.g., decoding) and poor response to effective instruction. However, Wagner et al.’s [6] model adds a feature of unexpectedness (e.g., unexpected impairment in sight word reading), which is found in definitions of dyslexia and supported by other researchers [1,7].

Although similarities can be seen between a clinical diagnosis and educational identification perspectives, differences complicate dyslexia classification in practice. Assessments used in dyslexia identification can vary based on the assessor and to which perspective they ascribe. For example, some assessors may include a measure of phonological skills (e.g., Comprehensive Test of Phonological Processing [8]) or spelling (e.g., Woodcock–Johnson Spelling subtest [9]). In contrast, others may only include standardized measures of reading fluency and accuracy (e.g., Gray Oral Reading Test [10]). See Table 1 for types of measures and examples used in diagnosing dyslexia. Differences are also seen across languages. Transparent languages (those with a one-to-one matching between sounds and spellings, such as Italian) are more likely to evaluate speed or rate of reading to determine a risk for dyslexia rather than accuracy of decoding, which is more likely to be assessed in less transparent languages, such as English [11]. Additionally, morphological awareness, in addition to phonological processing and rapid naming, has been found to be an important predictor of dyslexia in logographic languages, such as Chinese [12]. These differences will ultimately influence how dyslexia diagnosis is assigned. The complexity of variance in classification practices among clinicians and educators is a key challenge in the field as it results in heterogeneity among individuals identified with dyslexia.

## 2. Epidemiology of Dyslexia

**Figure 1:**
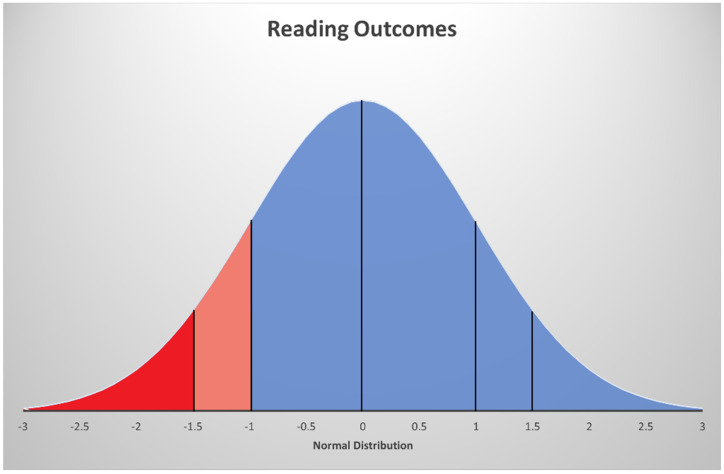
Comparison of categorical cut points in dyslexia definition. Note: red = cases of dyslexia (−1.5.SD or more below the mean are likely identified); orange = possible cases or controls (between −1 SD and −1.5 SD below the mean may be identified depending on the judgement of the assessor); blue = controls (identified as not having dyslexia).

Dyslexia is found worldwide and across languages, including transparent alphabetic orthographies, less transparent alphabetic orthographies, and logographic languages [14,15]. The prevalence of dyslexia varies in part due to the differences in definitions and criteria (e.g., unexpected poor performance vs. using patterns of strengths and weaknesses), the unreliability of some classification processes (e.g., determining response to effective instruction, including multi-tiered systems of support), and different levels of awareness by practitioners in recognizing and systematically assessing for dyslexia. Additionally, reading ability is continuously distributed across the population. Making a categorical determination (i.e., has dyslexia or does not have dyslexia) requires deciding where to place a cut point on this distribution, which, in turn, affects prevalence rates [6]. The DSM-5 suggests a −1.5 SD below the mean on standardized assessments is needed for recommended “diagnostic certainty” of a specific learning disability but allows for clinician judgement for −1 SD below the mean with other evidence to support the diagnosis [4] (p. 69). Categorizing dyslexia as present in those who score a −1.5 SD below the mean (approximately 7th percentile) on a reading measure vs. −1 SD below the mean (approximately 16th percentile) changes the prevalence rate of the disability (see Figure 1). These differences are reflected in the wide-range of dyslexia prevalence rates reported—between 3 and 17 percent [16,17], with most falling below 10 percent [18]. Prevalence rates fall within this range across a variety of studied languages, with slightly lower incidence rates found in more transparent languages, such as Spanish [19]. Dyslexia prevalence in studies of students who speak Chinese are also within a similar range (e.g., 2.3–8.4% [20]).

In addition, observations linked to biological risk factors have been reported across multiple studies. First, the prevalence of reading disability is greater in males than females [21]. Results from studies applying different diagnostic and identification criteria (e.g., low achievement in oral word reading) to reading impairment in population-wide samples suggest males display higher vulnerability rates than females. Sex odds ratios for reading difficulties increase with worsening severity of the reading problem. For example, for the relatively narrow measure of phonological decoding of nonsense words, the sex odds ratio peaked at approximately 1.6:1 for both low achievement and unexpected low achievement (i.e., aptitude–achievement discrepancy) operational criteria at the most severe level of reading impairment (i.e., below the 3rd percentile). Higher sex odds ratios were obtained for the broader measure of oral reading fluency, peaking at approximately 2.1:1 for the low achievement and 2.4:1 for the aptitude–achievement discrepancy criteria at the most severe level of reading impairment (i.e., 3rd percentile) [21]. Second, a family history of reading problems in a parent or sibling increases the risk of reading disabilities [17]. A study by Erbeli and colleagues [22] showed that the lower a child performed on a reading and spelling measure, the higher was her likelihood of having a positive family history of reading disability. Moreover, if either the mother or the father were affected as opposed to only the mother/only the father, a low performance on a reading or spelling measure was an even better indicator of a positive family history of reading problems. Finally, dyslexia can co-occur with other neurodevelopmental disorders, including attention-deficit/hyperactivity disorder (ADHD), a math disability, and/or developmental language disorder (DLD) [23,24]. In fact, ADHD symptoms or a math disability similarly co-occur in approximately 20 to 40% of individuals with reading difficulties [25]. The rates of comorbidity between dyslexia and DLD are even higher, with approximately 50 to 60% of individuals with one disability also meeting the criteria for the other [24]. These estimates are similar to those that are typically observed for most conditions of neurodevelopmental origin and consistent with a genetic etiology.

## 3. Twin Studies of Dyslexia

Abundant research over more than three decades using a twin design has provided convincing evidence that genetic factors underlie dyslexia [7,26,27]. Family studies have found that children of first-degree relatives with dyslexia have a higher risk for developing dyslexia as compared to relatives of controls (e.g., [28]). Mean prevalence (expressed as percentage) of dyslexia in the family-risk samples was 45% [17]. Twin studies have shown that the concordance rate for dyslexia in monozygotic twins (who are genetically identical) is increased compared to that of dizygotic twins (who are genetically no more similar than regular siblings), with most estimates lying between 40% and 70% [29,30]. In fact, a recent meta-analysis [31] across 49 twin studies in samples representative of a general population showed that reading abilities are strongly genetically influenced traits. For example, average heritability estimates for phonological awareness and rapid automatized naming (considered endophenotypes for dyslexia; e.g., [32]) are reported to lie at 46% and 52%, respectively. Average heritability estimates for letter–word knowledge, phonological decoding, reading comprehension, and reading are even higher (62–68%), with spelling displaying the highest average heritability estimate (80%) across all measured reading-related skills. However, for language skills, the same study reported a lower average heritability estimate (34%) [31]. Jointly, all these reports provide evidence that dyslexia owes strongly to genetic factors.

Factors such as age and sex can moderate heritability estimates from twin studies. Regarding age, in contrast to ADHD, where the heritability attenuates across adolescence and adulthood [33], genetic influences on dyslexia remain stable across adolescence and early adulthood [34]. Moreover, research has shown that the same genetic influences are manifested in childhood and early adulthood [34]. That means that the emergence of genetic factors, which would exert unique effects at different time points from childhood to adolescence and early adulthood, is less likely. A longitudinal assessment of 62 twin pairs with a history of reading disabilities and 77 twin pairs with no history of reading disabilities with a 5- to 6-year interval between assessments showed that both baseline deficits and the change in reading performance (e.g., a composite measure of reading) over time were primarily explained by genetic factors. Shared genetic influences accounted for 86% of the phenotypic correlation between reading assessments across two time points in twins with a history of reading disabilities [34]. Regarding the differential etiology of dyslexia as a function of sex, twin studies provide limited support for the claim. Although dyslexia prevalence is higher in males than females (see Section 2 on the epidemiology of dyslexia), twin studies using samples of over 1000 twin pairs suggest that genetic influences associated with dyslexia are no different for males and females, irrespective of dyslexia severity [31,35,36,37]. In other words, even though males might be more vulnerable, genetic etiology associated with dyslexia is similar in males and females.

In the last 15 years, twin studies studying dyslexia have started to move away from aspiring to achieve diagnostic homogeneity within dyslexia categories (i.e., has dyslexia or does not have dyslexia). Namely, this school of thought requires placing an arbitrary cutoff to diagnose affected individuals (see section above on the epidemiology of dyslexia and Figure 1, including the discussion on prevalence rates based on the chosen cut point). Based on this cutoff approach, the transformation of a continuous trait of reading performance into a categorical trait, which was reflected in the methods used in twin studies in the 1990s and early 2000s (e.g., probandwise concordance and deFries–Fulker analyses), resulted, by definition, in a loss of information pertaining to the continuum of variation in reading performance. Therefore, just as the knowledge about neurodevelopmental disorders progressed in the early 2000s, including the publishing of the generalist genes hypothesis in a seminal paper on generalist genes and learning disabilities [38], so did the behavioral genetics field start to address the shortcomings of a categorical designation of dyslexia. The field began to acknowledge that like essentially any other common neurodevelopmental disorder, dyslexia is heterogeneous at many levels, ranging from genetic risk factors to observable deficits across a normal distribution of reading skills. These advances were, in turn, reflected in the methodology used in subsequent twin studies, with samples representing the entire population rather than only a group of individuals with dyslexia, narrowly defined by an arbitrarily set cutoff. As such, findings from the latest twin studies support the idea that dyslexia is best viewed as the extreme end of a continuum of reading traits in the general population. For example, a study in 724 twin pairs showed that underlying genetic factors are associated with dyslexia symptoms above a typical identification threshold as well as below that threshold in the general population ([7]; see Figure 1 for a display of cut points in dyslexia definition across the continuum). While narrow categories might be useful for identifying underlying genes, they do not capture the clinical and educational reality of symptom heterogeneity across the entire continuum of reading ability.

Another complex issue about the categorical conceptualization of dyslexia is its widespread sharing of symptoms and risk factors with other childhood-onset neurodevelopmental disorders, such as dyscalculia, DLD, and ADHD. Accumulating evidence from twin studies suggests that genetic factors partly explain the comorbidity. For instance, a recent meta-analysis of 38 primary twin studies revealed an average genetic correlation between dyslexia and dyscalculia of 0.71 and dyslexia and ADHD of 0.42 [23]. These findings are in line with the generalized genes hypothesis [38], which states that most genes associated with learning disabilities in one area (e.g., reading disabilities) are likely associated with learning disabilities in another area (e.g., math disabilities). Even though these results imply strong genetic overlap across these neurodevelopmental disorders, they also suggest that each learning disability retains some unique genetic variance specific to a disorder, which is not shared with other disorders. Among later-onset disorders related to psychopathology and their comorbidity with dyslexia, the associations seem more complex. Regarding anxiety disorders (e.g., generalized anxiety disorder, stress disorders, panic disorder), while individuals with dyslexia are at an increased risk for this psychopathology, twin studies using large population samples (N = 1843 twin pairs) show that the association appears not to be overlapping due to genetic influences [39]. Regarding internalizing and externalizing behaviors, research shows genetic links with internalizing [40], whereas the relation between dyslexia and externalizing disorder owes primarily to shared environmental rather than genetic factors [40].

In summary, average estimates of heritability of reading-related skills and dyslexia, when conceptualized as a categorical disorder, are high (40–70%) and remain of similar magnitude across sex groups and development. Research in the last 15 years has consistently illustrated that common neurodevelopmental disorders, such as dyslexia, are the quantitative extreme of the same genetic factors responsible for the normal distribution of reading abilities. Moreover, genetic liability for dyslexia is shared with other neurobiological learning disorders (e.g., dyscalculia) and some psychopathological disorders (e.g., internalizing).

## 4. Molecular Genetics Studies of Dyslexia

The substantial heritability of dyslexia reported in twin studies motivated efforts to start conducting molecular genetics studies to identify specific genes associated with dyslexia. The first studies employed linkage analyses that highlighted a few chromosomal regions (named DYX1-9) likely to carry dyslexia susceptibility factors. Fine-scale mapping at the linked loci led to identifying associated variants within a few genes, including *DCDC2*, *KIAA0319*, *DYX1C1*, and *ROBO1*, which have been referred to for many years as “dyslexia candidate genes” (for extensive reviews, see [27,41,42]). Most of these studies were conducted in participants specifically selected for dyslexia. In these earlier studies efforts were made to exclude participants with comorbidities, assuming that a homogeneous dyslexia sample would facilitate the identification of genes contributing specifically to dyslexia.

The functional characterization of the identified genes and the observation of some of their common features led to theories aimed at explaining the neurobiology of dyslexia. Most notably, ‘knocked down’ experiments in rats suggested a role in neuronal migration for these genes [43]. In turn, these findings supported the idea that dyslexia results from defective neuronal migration as initially proposed by the Galaburda–Geschwind hypothesis [44,45,46]. The neuronal migration hypothesis remained prominent in the field until it was challenged by ‘knockout’ mouse models for some of the candidate genes (e.g., *KIAA0319*), which did not show the expected cortical anomalies (see [47] for a complete review of these studies).

Analysis of the cellular function of the candidate genes suggested an unexpected role in cilia, the sensory organelles that mediate many functions, including the mediation of extracellular stimuli [48,49]. Transcriptomic analysis showed that dyslexia-associated genes are upregulated in ciliated tissues [50]. Both knockdown and knockout cellular and animal models provided evidence for a role in cilia formation and cilia length regulation of *KIAA0319, DCDC2*, and *DYX1C1* [51,52,53]. Mutations of both *DYX1C1* and *DCDC2* have been found in patients with primary ciliary dyskinesia and nephronophthisis-related ciliopathies, respectively [53,54]. Carriers of these mutations presented severe organ dysfunctions caused by defective cilia but did not present any symptoms of dyslexia. Therefore, while research on these genes have highlighted interesting mechanisms underlying neurodevelopment, they cannot be linked directly to dyslexia.

Beyond the role and function of individual genes, as much as convincing in developing a narrative that would fit a credible theory, it is worth noting that the original studies that implicated them were conducted in small samples—up to a few hundred participants. Hence, it is not surprising that studies using larger sample sizes consistently failed to replicate these initial associations [55,56,57]. As the field of complex trait genetics progresses, we learn that complex neurodevelopmental disorders, such as dyslexia, have a highly polygenic nature where many individual genetic factors contribute minimal effects to a phenotype. The detection of such effects requires genome-wide approaches using large sample sizes.

### 4.1. GWAS: The Sample Size Problem

Genome wide-association studies (GWASs) [58] are the gold standard method for identifying genetic factors associated with complex traits, such as dyslexia. GWASs involve analyses of many single-nucleotide polymorphisms (SNPs), which are spread across the genome. The success of GWASs is determined mainly by the sample size employed. Sufficiently large samples can be obtained through extensive international collaborations or thanks to resources such as the UK Biobank (https://www.ukbiobank.ac.uk/, accessed on 20 December 2021), which gives access to hundreds of phenotypes and genomic data for half a million people [59].

Nonetheless, collecting sufficiently powered samples for dyslexia GWAS has been challenging. As a result, progress in conducting GWAS research for dyslexia has not been made at the same pace as for other traits and disorders. As mentioned above, some of the key challenges are represented by the different criteria used to assess reading skills and dyslexia identification, especially when considering cross-national projects. Moreover, dyslexia is not screened for systematically via national health systems. Therefore, the phenotypic data available via large cohorts such as the UK Biobank are not ideal. Dyslexia identification in large population cohorts tends to rely on self-report, which will suffer from heterogeneity and biases. Furthermore, most large cohorts are based on adult participants who were children when dyslexia awareness was lower, most likely having resulted in misidentification.

This challenge can, in part, be circumvented by using birth cohorts that collect regular cognitive and behavioral assessments at critical developmental milestones. Two British cohorts, the Avon Longitudinal Study of Parents and Children (ALSPAC) cohort and the Twins Early Development Study (TEDS), use the batteries of tests similar to those employed for dyslexia identification (e.g., single-word reading; see Table 1). However, the high-quality assessments are limited to several thousands of participants, sample sizes much smaller in comparison to adult cohorts such as the UK Biobank.

One way forward to increase sample sizes is to aggregate data across multiple cohorts. International initiatives, such as NeuroDys, with a primary focus on dyslexia, and GenLang, focusing on language-related disorders, more generally, have tackled this very problem. They have developed platforms for both data sharing and data harmonization for cross-linguistic settings, leading to the first exciting breakthroughs, as will be described in the next section.

### 4.2. GWAS for Reading Skills and Dyslexia

Typically, GWASs are conducted using a case–control design. However, some of the challenges discussed above mean that sufficiently large samples meeting a dyslexia diagnosis as well as controls assessed using identical criteria has been an arduous undertaking for a long time. Therefore, population-based cohorts characterized by reading measures have been used to identify individuals at the extremes of reading ability distribution, enabling researchers to obtain sufficient sample sizes for the case/control categories. For example, an initial GWAS conducted in the TEDS samples compared poor and good readers (about 750 individuals in each group; see Figure 1) [60].

Another strategy is to conduct GWASs using a quantitative model. This strategy takes advantage of the entire distribution of reading abilities and allows us to find genetic associations for individual differences in reading [41]. This strategy was used in a subsequent study in about 3000 TEDS twin pairs [61]. Neither of these TEDS studies found any statistically significant association. However, the study by Davis and colleagues [61] confirmed a strong heritability for reading abilities based on molecular data. Furthermore, the findings showed that shared genetic effects could explain around 50% of the correlation between reading and math, a finding consistent with results from a twin-based meta-analysis (see Section 3 on twin studies of dyslexia) [23].

Other quantitative GWASs in population cohorts have analyzed reading together with language measures, because of the high comorbidity rates between dyslexia and DLD. These included analyses of around 5000 ALSPAC and 1177 Australian participants [62]. A subsequent GWAS was conducted in individuals selected for reading and language difficulties, including NeuroDys participants (N = 1862) [63]. The most interesting findings from these studies were associations that clustered around the *CCDC136* and *FLNC* genes for both reading and language measures. A more recent study, using a quantitative approach in a larger NeuroDys sample, reported a significant association (*p* = 4.73 × 10^−9^) of the *MIR924HG* (micro-RNA 924 host gene) gene with RAN [64]. A different GWAS was explicitly conducted for RAN in a sample of Hispanic Americans and African Americans (N = 1331), reporting an association at the *RPL7P34* gene [65]. The latest GWAS for continuous reading and language measures was conducted in 34,000 participants from the GenLang project [66]. The measures included word reading, nonword reading, spelling, phonemic awareness, and nonword repetition. Despite a large sample size, only one statistically significant association was detected. Specifically, the association was observed between a SNP on chromosome 1 (rs11208009) and word reading. Beyond the specific marker–trait association, all five measures presented substantial SNP-heritability estimates (0.13–0.26). Genomic structural equation modelling showed that most variation in reading, spelling, and phonemic awareness was owed to shared genetic factors, which is consistent with the findings from twin studies (e.g., [26,67]). However, individual differences associated with genetic factors in these three skills only partially overlapped with variability in nonword repetition and general cognition. The study also found a correlation between genetics contributing to individual differences in the five measures and cortical surface areas known to process language functions. The genetic associations tended to occur in genomic regions that appear to have had a critical role in the evolution of modern humans.

Only recently, sufficiently large samples have been achieved for case–control GWAS using a categorical definition of dyslexia. A GWAS conducted in the NeuroDys samples compared 2274 cases and 6272 phenotyped controls under a case–control model [57]. The study estimated that up to 20–25% of dyslexia susceptibility could be explained by common genetic variants—a finding similar to that observed in the above mentioned quantitative GWAS for reading and language measures by Eising and colleagues [66]. The latest and largest case–control GWAS study was conducted in 51,800 cases and 1,087,070 controls. Dyslexia diagnosis relied on a self-report [56]. Such a large sample set was obtainable by accessing data from the direct-to-consumers 23andMe database (https://www.23andme.com/, accessed on 20 December 2021). Although the binary categories were defined through a very crude measure (i.e., a yes/no answer to the question, “Have you ever been diagnosed with dyslexia?”), the large sample size led to the identification of 42 statistically significant associations. Of these, 17 were previously reported to be associated with cognitive abilities and educational attainment, supporting the idea of generalized effects. However, 17 associations appeared to be specific to dyslexia. About half of the 42 associations, including 12 of the dyslexia-specific loci, were replicated later in independent cohorts of Chinese and European ancestry. The study revealed high genetic correlations (−0.45 < *r_g_* < −0.75) with the reading and language measures of the GenLang cohort [66]. Such a finding provides strong evidence that the genetic determinates of a clinical dyslexia diagnosis correlate with the genetics contributing to individual differences in reading abilities (i.e., a continuous distribution; Figure 1). A high genetic correlation (0.53) was also found for ADHD, but no correlation was found for neuroanatomical measures of language-related circuitry. Finally, this large GWAS also allowed for generating reliable polygenic risk scores (PRSs) for dyslexia, which have been validated in independent cohorts, including the GenLang samples [64], explaining up to 6% of the variance in reading outcomes.

### 4.3. Polygenic Risk Scores

PRSs aggregate the effects of multiple molecular genetic markers (SNPs) to assign to each individual a score that predicts the risk for a trait or disorder [68]. To be reliable, PRSs require to be generated from very large GWASs. Although the main goal of PRSs analysis is to assign a risk score to each person, they can only be used to test whether the genes associated with a particular trait also influence other phenotypes. PRSs for a range of neurodevelopmental traits have been tested for their association with dyslexia and reading abilities.

PRSs for educational attainment [69] accounted for up to 5.1% of the variance in reading measures at the age of 14 in the TED sample [70]. This result has since then been replicated in subsequent studies (e.g., [54,68,71]). For example, in the NeuroDys samples, PRSs for educational attainment explained almost 2% of the total variance in reading abilities, including spelling, non-word reading, phonological awareness, and digit span [64]. In terms of PRSs for other cognitive traits and psychiatric disorders, PRSs for intelligence have shown stronger associations, compared to educational attainment, for both dyslexia (*p*_Intelligence_ = 9.4 × 10^−29^; *p*_EA_ = 1.95 × 10^−7^) and reading abilities (*p*_Intelligence_ = 7.25 × 10^−181^*; p*_EA_ = 4.91 × 10^−48^). Both studies also detected significant associations for ADHD and bipolar disorder PRSs. In particular, Gialluisi and colleagues [57] found that bipolar disorder PRSs had a stronger association than ADHD PRSs (*p_Bipolar_* = 1.33 × 10^−43^; *p*_ADHD_ = 7.66 × 10^−13^) and explained a larger proportion (2.8% vs. 0.7%) of the variance in dyslexia risk. PRSs for schizophrenia also showed a significant association with dyslexia risk (*p* = 3.65 × 10^−22^) and explained 1.4% of the variance.

In sum, two key observations can be derived from these findings. First, the similar patterns observed for dyslexia and reading abilities support the idea that the same genetic factors contributing to variation in general reading abilities also influence dyslexia when conceptualized as a categorical disorder, as illustrated in Figure 1. Second, some of the factors contributing to dyslexia and reading abilities also have an effect on a range of neurodevelopment disorders and traits. While this is expected to be the case for disorders co-occurring with dyslexia, such as ADHD, it is surprising to observe more substantial effects for conditions such as bipolar disorder and schizophrenia, which are clinically distinct from dyslexia. Hence, it appears generic genetic backgrounds serve as risk or protective factors for various neurodevelopmental disorders with distinct clinical manifestations. In addition to what was found, it is also important to note what these studies did not find. Remarkably, the original associations in the “dyslexia candidate genes” reported in early molecular genetic studies failed to be detected in any GWASs, strongly suggesting they were false positives.

### 4.4. Rare Variants

While the results reported thus far confirm a strong polygenic nature of dyslexia, large effects associated with individual variants remain a possibility. Rare variants for dyslexia have not been assessed systematically in large samples. Nonetheless, a few examples have been reported. *DYX1C1*, the first gene to be implicated in dyslexia, was identified by mapping a translocation breakpoint at 15q21 [72]. Other large deletions/insertions at chromosome 15 (15q11.2) were found to be associated with reading abilities and dyslexia in the deCODE Icelandic cohort (N > 100,000) [73,74]. Rearrangements at the chromosome 15q locus have been reported in cases with seizures and severe intellectual disability; however, a large de novo deletion 15q13 was reported in a child with language impairments but no other deficits [75]. Therefore, it appears that the 15q locus is associated with a range of neurodevelopmental phenotypes with a variable degree of severity, including specific effects on reading/language difficulties. A similar scenario was reported for a particular rare variant at the *ATP2C2* gene on chromosome 16 identified in an individual with language impairment. Follow-up analyses in the general population found that exactly that same variant was associated with poor performance in reading skills [76]. Such a pattern was observed also for a variant in the 3′ UTR of *SPRY1,* which co-segregated with dyslexia in one family. Other variants in the same region also showed association with short-term memory in a larger sample of about 2500 individuals [77]. It is possible that the range of effects observed at the phenotypic level for a particular variant resulted from the interaction with different polygenic backgrounds. For example, an individual with the *ATP2C2* risk variants might develop DLD when also carrying a high-risk PRS for language problems. While this variant is not sufficient in and of itself to lead to a diagnosis, at a group level, it is associated with poor performance in reading tasks, regardless of the polygenic backgrounds.

Overall, it is essential that more data be collected to evaluate the effects of rare variants in dyslexia. The decrease in the cost of sequencing technology will facilitate such efforts.

## 5. Conclusions

For at least four decades, genetic underpinnings of dyslexia, a reading disability of neurobiological origin, have been studied. Genetic research in the last two decades has led to significant advances in our understanding of dyslexia. Both twin and molecular genetic studies have converged to the idea that dyslexia represents a lower tail of normally distributed reading abilities. What exact diagnosis and identification criteria we implement and when and where we set a categorical cut point remains debated, though. This demonstrates the challenges that remain of how to define dyslexia unequivocally within the genetics field. Regardless, a categorical definition of dyslexia remains useful, in particular in helping achieve large sample sizes required by GWASs.

The complexity of the phenotypic definition mirrors the high polygenicity of dyslexia. At the beginning of genetic dyslexia research, it was predicted that only a few genes were associated with the disorder. In fact, it was expected that it might have even been possible to identify specific genes–specific endophenotypes correspondences. As research progressed, we have started to appreciate a large number of genes involved in the variability of neurodevelopmental phenotypes, including dyslexia, further demonstrating how blurry the categories are not only between dyslexia definitions but also across clinically distinct disorders. Until 10 years ago, the main bottleneck was represented by our ability to generate genomic data. With human genomes generated rapidly and at an affordable cost, this is no longer an issue. The new challenge is now the collection of high-quality quantitative measures that can meaningfully capture variability among people, especially in cross-linguistic settings. Analyses similar to those described in the current report but applied to ethnicities other than white Europeans will contribute to our understanding of dyslexia neurobiology, by investigating similarities and differences across populations and their written and spoken languages. Online tests are one way to provide an innovative platform for such an undertaking. Combining such approaches with genomic screenings in tens of thousands of people is likely to lead to significant discoveries and make the next 10 years even more exciting than the past 20 ones.

## Figures and Tables

**Table 1:** Assessments commonly used in identifying dyslexia [13].

Construct Measured	Description	Examples
Word Recognition	Ability to read single words or sight words	Wechsler Individual Achievement Test—Word Reading
Decoding	Ability to read unfamiliar words using letter–sound knowledge (often nonsense words)	Wechsler Individual Achievement Test—Decoding
Spelling	Ability to spell words from memory	Woodcock–Johnson Spelling
Phonological Processing	Ability to identify, pronounce, or recall individual sounds (phonemes)	Comprehensive Test of Phonological Processing
Fluency	Ability to read word lists or passages at an appropriate pace	Gray Oral Reading Test
Rapid Automatized Naming	Ability to quickly name a series of objects, colors, numbers, or letters	Comprehensive Test of Phonological Processing
Reading Comprehension	Ability to understand and answer questions about an independently read text	Woodcock–Johnson Reading Comprehension
Listening Comprehension or Oral Language	Ability to understand a story read-aloud or spoken directions	Wechsler Individual Achievement Test—Oral Language

## Data Availability

Not applicable.
